# Up-Cycling Broccoli Stalks into Fresh-Cut Sticks: Postharvest Strategies for Quality and Shelf-Life Enhancement

**DOI:** 10.3390/foods14142476

**Published:** 2025-07-15

**Authors:** Nieves García-Lorca, José Ángel Salas-Millán, Encarna Aguayo

**Affiliations:** 1 Postharvest and Refrigeration Group, Universidad Politécnica de Cartagena (UPCT), Paseo Alfonso XIII, 48, 30203 Cartagena, Spain; nieves.garcial@upct.es; 2 Food Quality and Health Group, Institute of Plant Biotechnology (IBV-UPCT), Campus Muralla Del Mar, R&D Building, 30202 Cartagena, Spain; joseangel.salas@upct.es

**Keywords:** calcium ascorbate, hot water treatment, trehalose, glucosinolates, circular economy, revalorisation

## Abstract

Broccoli stalks are considered an agro-industrial by-product that, in the context of fresh consumption, is undervalued, as only broccoli florets are typically marketed. This study evaluated the up-cycling of broccoli stalks into a value-added fresh-cut product through postharvest preservation strategies. Stalks were peeled, cut into sticks (8 × 8 mm × 50–100 mm), sanitised, packaged under modified atmosphere conditions, and stored at 5 °C. Treatments included (a) calcium ascorbate (CaAsc, 1% *w*/*v*), (b) trehalose (TREH, 5% *w*/*v*), (c) hot water treatment (HWT, 55 °C, 1 min), and several combinations of them. HWT alone was highly effective in reducing browning, a key factor for achieving an extended shelf-life, controlling microbial growth and respiration, and obtaining the highest sensory scores (appearance = 7.3 on day 11). However, it was less effective in preserving bioactive compounds. The HWT + CaAsc treatment proved to be the most effective at optimising quality and retaining health-promoting compounds. It increased vitamin C retention by 78%, antioxidant capacity by 68%, and total phenolic content by 65% compared to the control on day 11. This synergistic effect was attributed to the antioxidant action of ascorbic acid in CaAsc. TREH alone showed no preservative effect, inducing browning, elevated respiration, and microbial proliferation. Overall, combining mild thermal and antioxidant treatments offers a promising strategy to valorise broccoli stalks as fresh-cut snacks. An 11-day shelf-life at 5 °C was achieved, with increased content of health-promoting bioactive compounds, while supporting circular economy principles and contributing to food loss mitigation.

## 1. Introduction

Broccoli *(Brassica oleracea* L.) is a cruciferous vegetable widely appreciated for its notable nutritional profile and health-promoting bioactive compounds, such as glucosinolates (GSLs), phenolic compounds, and vitamin C [1,2]. These phytochemicals exhibit antioxidant, anti-inflammatory, and chemopreventive effects, contributing to the recognition of broccoli as a functional food [3]. However, the commercial exploitation of broccoli is predominantly limited to its florets, which constitute only around 15% of the total biomass, whereas the stalks (21%), leaves (47%), and roots (17%) are commonly discarded, resulting in a significant underutilisation of valuable plant tissue [4,5].

Broccoli stalks are particularly rich in dietary fibre, GSLs, and residual sugars, positioning them as a promising raw material for the development of functional foods [6]. Nonetheless, their high-water activity and elevated respiration rates make them highly perishable, and they are prone to enzymatic browning, microbial spoilage, and nutrient degradation during storage [7,8]. These challenges are further exacerbated during minimal processing, where cutting, peeling, and washing lead to the disruption of cellular compartments, the activation of oxidative enzymes, and an increased microbial load [9]. In this context, transforming broccoli stalks into a fresh-cut, minimally processed, ready-to-eat product represents a novel strategy for the valorisation of agro-industrial by-products. Fresh-cut products are fresh fruits or vegetables that have been physically altered from their original form, such as by cutting, slicing, dicing, or peeling, while remaining in a fresh, raw state. These products are typically washed, minimally processed, refrigerated, and packaged to ensure safety and to extend shelf life while preserving their freshness [10]. This approach not only supports circular economy principles through waste reduction and resource efficiency but also offers a nutritious and practical alternative for consumers. Broccoli sticks could be marketed in a similar way to carrot or celery sticks, which are suitable for inclusion in salad bars and individual packs or as convenient ready-to-eat options for dipping. This innovation responds to the growing consumer demand for sustainable, health-oriented vegetable-based products, as reflected in the expansion of the global healthy snacks market, which was projected to reach USD 107 billion by 2024, with a CAGR of approximately 6.4% [11].

Of the different postharvest treatments available, antioxidant treatments, osmoprotectants, and mild thermal processes are among the most effective strategies for maintaining postharvest quality in fresh-cut produce. Calcium ascorbate (CaAsc), a salt formed by calcium and ascorbic acid, combines the antioxidant effect of ascorbic acid with the structural reinforcement conferred by calcium [12]. Ascorbic acid acts as a reducing agent that inhibits polyphenol oxidase (PPO)-mediated browning by reducing o-quinones to their precursor diphenols, thereby preventing the formation of melanins [13]. Simultaneously, Ca^2+^ ions reinforce the middle lamella and maintain firmness, slowing enzymatic degradation and limiting microbial infiltration [14,15]. CaAsc has demonstrated efficacy in preserving vitamin C, total phenolic content (TPC), and antioxidant capacity during cold storage in products such as melon [16], apple [17], and potato [18].

Trehalose (TREH), a non-reducing disaccharide, has attracted attention as an osmoprotectant and stress mitigator in postharvest physiology. It stabilises membranes and proteins under abiotic stress, which reduces oxidative damage and maintains tissue turgor [19,20]. Its application to fruits such as guava [21], cherry tomato [22], kiwifruit [23], and litchi [24] has delayed postharvest browning and improved membrane stability, antioxidant enzyme activity, and water retention. However, the effects of TREH are highly species- and matrix-dependent; thus, its effectiveness in broccoli cannot be assumed merely based on results from other crops. For instance, it may alter microbial dynamics in broccoli, due to its bioavailability as a carbon source for certain spoilage microorganisms [25], and may modulate PPO and peroxidase (POD) activity in unpredictable ways, potentially intensifying browning reactions [26]. Therefore, its effectiveness as a preservative must be validated for each target product.

Hot water treatment (HWT) is a traditional postharvest tool that is gaining relevance as a non-chemical preservation method that combines efficacy and consumer acceptance. HWT involves immersing the product in water at sublethal temperatures (typically 45–55 °C) for a short period of time (30 s to 2 min), thereby triggering the partial denaturation of degradative enzymes such as PPO, stimulating heat shock responses, and suppressing initial microbial loads [27,28]. HWT has been shown to delay senescence, reduce the respiration rate, and enhance the retention of antioxidant compounds during storage in fresh-cut apples, melons, and Chinese cabbage [12,29]. Moreover, HWT facilitates the uptake of solutes into plant tissues by increasing membrane permeability and promoting tissue porosity [30], which could enhance the efficacy of subsequent antioxidant dips such as CaAsc.

While these technologies have been widely studied in fresh-cut vegetables, the postharvest physiology and preservation of broccoli stalks remain unexplored. Their distinct anatomy and chemical composition, particularly the high concentrations of GSLs in outer tissues [31], may influence the efficacy of common preservation treatments. Previous studies have also shown that GSL profiles are sensitive to both enzymatic hydrolysis and thermal degradation [32,33], and thus require tailored strategies to minimise nutrient loss.

Therefore, the objective of the present study is to assess the individual and combined effects of CaAsc, TREH, and HWT, integrated with modified atmosphere packaging (MAP), on the shelf-life, microbial quality, sensory attributes, and bioactive compound retention in fresh-cut broccoli stalk sticks. This research seeks to establish an optimised postharvest protocol that enhances the valorisation of broccoli by-products and supports sustainable innovation in the fresh-cut vegetable industry.

## 2. Materials and Methods

### 2.1. Plant Material and Treatments

Fresh broccoli stalks (*Brassica oleracea* L. var. *Italica*, cv. Parthenon) are an undervalued by-product in the context of fresh consumption, as only the broccoli florets are typically marketed, while the stalks are largely discarded. Broccoli stalks were obtained from Levante Sur Coop. in the southeast of Spain (La Palma, Cartagena, Spain). Firstly, to obtain broccoli sticks, the broccoli stalks were washed with tap water to remove any dust and dirt. Subsequently, they were preliminarily disinfected by immersion in an 80 ppm peroxyacetic acid (PAA) solution for one minute. PAA was chosen because of its proven antimicrobial efficacy and its approval as a technological coadjuvant for washing fruits and vegetables, as recognised by the Spanish Food Safety Authority (AESAN) [34]. After being disinfected, the broccoli stalks were manually peeled with a knife and then cut into sticks measuring 8 × 8 mm in width and 50–100 mm in length with a vegetable stick cutter (model QTJ-J002X, VEVOR, Shanghai, China). Following the cutting process, the broccoli sticks underwent a second disinfection step in an 80 ppm peroxyacetic acid solution for one minute. Thereafter, the broccoli sticks were subjected to seven different treatments and also a control group that received no additional treatment beyond the disinfection procedure.

The treatments applied to the broccoli sticks were as follows: (a) CaAsc: the broccoli sticks were immersed in a cooled (5 °C) 1% (*w*/*v*) CaAsc solution for one min. (b) TREH: sticks were submerged in a cooled 5% (*w*/*v*) TREH solution for one min. (c) HWT: sticks were submerged for one min in water at 55 °C, followed by immediate cooling in a cold-water bath (5 °C). In addition to these three treatments, this study evaluated a further four combinations of the above treatments: (d) CaAsc + TREH: the sticks were sequentially immersed in a 1% CaAsc solution followed by a 5% TREH solution, each for one min and at 5 °C. (e) HWT + CaAsc: sticks were immersed in hot water at 55 °C for one min, then immediately cooled in a 1% CaAsc solution for a further min. (f) HWT + TREH: sticks were immersed in hot water at 55 °C for one min, followed by cooling in a 5% TREH solution for one min. (g) HWT + CaAsc + TREH: sticks were sequentially treated in hot water at 55 °C for one min, then cooled in both 1% CaAsc as well as 5% TREH solutions, each for one min. After the treatments, 120 g of broccoli sticks were packed in 500 mL trays and sealed with polypropylene film (microperforated Pplus, Amcor Plc, Zürich, Switzerland) for MAP, helping to maintain high relative humidity within the package. The trays were stored in a cold storage chamber (5 ± 1 °C), which was kept dark throughout the storage period (11 days), except for when the lights were briefly switched-on during sample collection, which was carried out on days 0, 4, 8, and 11. A minimum of three replicates (trays) per treatment were prepared.

### 2.2. Browning Index

Surface colour measurements were performed on 10 broccoli sticks per treatment and storage day using a Minolta CR-400 colorimeter (Minolta Inc., Tokyo, Japan). The device was calibrated with a white porcelain plate before use. For each broccoli stick, three random points were measured. Chromatic values were determined according to the CIELAB system and recorded as L* (lightness), a* (red–green component), and b* (yellow–blue component). The browning index (*BI*) was calculated following the method described by Kasim and Kasim [35], using Equation (1).

(1) $$BI=\frac{100\;(x-0.31)}{0.17}\;\;\mathrm{where}\;x=\frac{\left(a^{*}+1.75\;L^{*}\right)}{\left(5.645\;L^{*}+\;a^{*}-\;0.012\;b^{*}\right)}$$

### 2.3. Microbiological Analysis

Microbiological analysis was conducted following standard methods to evaluate the microbial quality of the broccoli sticks. Samples were prepared by homogenising 10 g of stick sections from at least three different sticks per tray and treating in 90 mL of sterile peptone-buffered water for one min using a 400 Lab Stomacher (Seward Medical, London, UK). As described by Martínez-Hernández et al. [36], peptone-buffered water serves as an effective diluent for serial dilutions, ensuring accurate microbial enumeration. Serial dilutions were prepared and plated on selective media (Scharlau Chemie S.A., Barcelona, Spain) to quantify microbial groups under specific incubation conditions: mesophilic aerobic bacteria were plated on Plate Count Agar and incubated at 30 °C for 72 h; enterobacteria were plated on Violet Red Bile Dextrose Agar and incubated at 37 °C for 24 h; yeasts and moulds were plated on Rose Bengal Chloramphenicol Agar and incubated at 25 °C for 96 h; and pathogenic bacteria: *Salmonella* spp. was assessed using Xylose Lysine Deoxycholate Agar, while *Escherichia coli* was quantified using Tryptone Bile X-glucuronide Agar. All microbial counts were conducted in triplicate and expressed as log CFU/g where CFU (Colony-Forming Unit) represents the number of viable microorganisms capable of forming colonies under the given incubation conditions.

### 2.4. Gas Composition Analysis

The internal atmospheric composition in the trays was regularly monitored using a CheckPoint gas analyser (PCE Ibérica, S.L., Tobarra, Spain). For the analysis, a 25 mL gas sample was taken from each tray through a silicone septum, allowing the measurement of oxygen (O_2_) and carbon dioxide (CO_2_) levels. The device was calibrated between each set of measurements by sampling the ambient air in the room to ensure accuracy. Gas composition was measured in triplicate for each tray and treatment on specific days (0, 4, 8, and 11) of the product’s shelf-life.

### 2.5. Sensory Analysis

The sensory evaluation was conducted by a trained panel of 10 individuals in a controlled sensory laboratory, where each panellist was seated in an individual booth to control for external factors and to ensure an objective sensory assessment. Water was provided for palate cleansing between samples, and each panellist was presented with three broccoli sticks per treatment, coded with random letters to prevent bias. Sensory analysis included evaluations of browning and dehydration using a 9-point scale, where 1 indicated very low quality (e.g., very brown or severely dehydrated) and 9 indicated optimal quality (no browning or fully hydrated). Appearance, firmness, crispness, and taste acceptability were assessed on a 9-point hedonic scale, in which 1 meant “dislike very much,” 9 “like very much,” and 5 represented the threshold for marketability. Finally, overall acceptability was rated by integrating all assessed attributes, also using the same 9-point scale, where scores ≥ 5 indicated potential consumer acceptance.

This trained sensory evaluation did not require an ethical statement, since it involved no invasive procedures or health-related interventions. Before starting the evaluation, the research team explained the scope and details of the project to the participants, including the purpose of the research, the identity of the researchers, data protection measures, privacy and data retention policies, the voluntary nature of participation, the right to withdraw at any time, and contact details for any questions that might arise. Finally, all participants signed a written informed consent form, confirming that they had read and understood the information provided, and that their questions had been answered.

### 2.6. Chemical Analysis

For the chemical analysis described below, the broccoli sticks were cooled, freeze-dried, ground into a fine powder, and stored at room temperature in vacuum-sealed bags until analysis.

#### 2.6.1. Vitamin C Content

Vitamin C analysis was performed using High-Performance Liquid Chromatography (HPLC) as previously described by Rasines et al. [37] with slight modifications. A 0.5 g sample of freeze-dried broccoli stick powder was homogenised with 10 mL of cold extraction buffer (0.1 M citric acid, 0.05% ethylenediaminetetraacetic acid (EDTA), 4 mM sodium fluoride, and 5% methanol) using an Ultra-Turrax^®^ homogeniser (IKA, Berlin, Germany). The mixture was kept on ice and in darkness. The homogenate was then filtered through a 4-layer gauze, and the pH of the extract was rapidly adjusted to 2.35–2.40 using 1 M HCl. Subsequently, 2 mL of the sample was centrifuged at 10,500× *g* for 5 min at 4 °C. The resulting supernatant was purified using Sep-Pak C18 cartridges (Waters, Dublin, Ireland), which were previously activated with 10 mL methanol, 10 mL ultrapure water, and 10 mL air. The purified extract was then filtered through a 0.45 μm nylon filter. A 750 μL aliquot of the filtered extract was derivatised with 250 μL 1,2-phenylenediamine (OPDA) and incubated at room temperature for 37 min. Afterwards, 20 μL of the derivatised extract was analysed by HPLC (1100 series, Agilent Technologies, Waldbronn, Germany), equipped with a G1322A degasser, G1311A quaternary pump, G1313A autosampler, G1316A column heater, and G1315B photodiode array detector. The chromatographic separation was carried out under isocratic conditions using a mobile phase consisting of 5 mM hexadecyltrimethylammonium bromide (CTAB) and 50 mM potassium dihydrogen phosphate in methanol/ultrapure water (5:95, *v*/*v*) at a flow rate of 1.6 mL min^−1^. Chromatograms were recorded at 261 nm for ascorbic acid and 348 nm for dehydroascorbic acid (Merck-Sigma, Darmstadt, Germany). Each of the three replicates was analysed in triplicate. Vitamin C content was quantified as the sum of ascorbic acid and dehydroascorbic acid, using authentic standards for their identification, and the results were expressed as mg 100 g^−1^ dry weight (DW).

#### 2.6.2. Antioxidant Capacity and TPC

Antioxidant capacity and TPC were determined in freeze-dried broccoli stick samples. For extraction, 0.2 g of the freeze-dried sample was mixed with 15 mL of methanol/water solution (70:30, *v*/*v*) and subjected to ultrasonication to enhance extraction efficiency. The sample was then vortexed for 30 s to ensure homogenisation. After centrifugation, the supernatant was filtered and collected. Three repetitions were performed and analysed for each treatment and storage day.

The antioxidant capacity was measured using the ABTS assay, following the method described by Re et al. [38] with slight modifications. The ABTS·^+^ radical was generated by dissolving 10 mg of ABTS reagent (2,2′-azinobis-(3-ethylbenzothiazoline-6-sulfonate)) in a potassium persulfate solution, followed by incubation in the dark for 12–24 h. Once the radical was formed, the ABTS·^+^ solution was diluted and adjusted to an absorbance of 0.7 ± 0.02 at 734 nm to establish a baseline. A stock solution of Trolox (Sigma Aldrich, St. Louis, MO, USA) was prepared in ethanol and diluted to different concentrations (5–100 μM) to generate a calibration curve. The absorbance was measured at 734 nm, and the curve was obtained by plotting absorbance values against Trolox concentrations. For the assay, 300 μL of the adjusted ABTS·^+^ solution was added to each well of a microplate, and an initial absorbance reading was taken. Then, 30 μL of water (for blanks), Trolox standard, or sample extract was added to the wells. After a 3 min incubation, the absorbance was measured again. The decrease in absorbance reflected the antioxidant capacity of the samples, which was expressed as milligrams of Trolox equivalents per gram of DW (mg TE g^−1^ DW), based on the calibration curve.

The TPC assay was determined using a multiscan plate reader (Tecan Infinite M200, Männedorf, Switzerland), following the method outlined by Salas-Millán et al. [39], with slight modifications. A volume of 20 μL from the previously extracted sample was pipetted into each well of a microplate, followed by 30 μL of Folin–Ciocalteu reagent (diluted 1:1 with ultra-distilled water) and 200 μL of a sodium hydroxide/sodium carbonate (NaOH/Na_2_CO_3_) mixture. The microplate was then incubated for 40 min at room temperature in the dark. Prior to measurement, the microplate was automatically shaken, and absorbance was recorded at 765 nm. TPC was expressed as milligrams of gallic acid equivalents per gram of DW (mg GAE g^−1^ DW).

#### 2.6.3. Identification and Quantification of GSLs

The identification and quantification of GSLs were performed following the method described by Salas-Millán et al. [32], with slight modifications. Freeze-dried samples (0.1 g) were extracted using 5 mL of methanol/water (70:30, *v*/*v*). The extraction process involved incubation in a hot bath at 70 °C, followed by centrifugation and filtration. A 3 mL aliquot of the supernatant was then evaporated under nitrogen gas and reconstituted in 1 mL of mobile phase A. The mobile phase consisted of 0.1% formic acid in water (A) and 0.1% formic acid in acetonitrile (B). The analysis was performed in the negative ionisation mode using HPLC (Agilent 1200, Santa Clara, CA, USA), equipped with a G1311B quaternary pump, G1329B standard autosampler, and G1316A column heater, and coupled to a 6420 triple–quadrupole mass spectrometer (QqQ) with an electrospray ionisation (ESI) source. The autosampler was programmed to inject 10 μL of the eluted sample at a flow rate of 0.250 mL min^−1^. The chromatographic gradient was set as follows: 5–30% B (14 min), 30–95% B (7 min), and 95–95% B (5 min), at 40 °C. For the identification of GSLs, the retention time, mass (MS), and MS/MS fragmentation spectra of the detected compounds were compared with a commercial standard or tentatively identified by comparing their fragmentation patterns with the available bibliographic data and the MassBank Europe, MassBank of North America, and PubChem databases. Calibration curves were generated using standards of each GSLs, which were used to quantify their concentrations. Finally, the results were expressed as mg 100 g^−1^ DW.

### 2.7. Statistical Analysis

Statistical analyses were conducted using Statgraphics Centurion XV, version 15.2.05 (Statgraphics Technologies, Inc., The Plains, VA, USA). A two-way analysis of variance (ANOVA) was conducted to evaluate the effect of postharvest treatments (T) and days of storage time (D) on each dependent variable (*p* < 0.05). Results were expressed as mean values ± standard error (SE). When significant differences were found (*p* < 0.05), Fisher’s Least Significant Difference (LSD) test was used for multiple comparisons. A cluster correlation heatmap was generated using Ward’s hierarchical clustering method and Euclidean distance. Ward’s method was chosen as it minimises the total within-cluster variance, generating more compact and interpretable clusters for multivariate datasets such as those obtained in postharvest studies. Before the analysis, the data were normalised using the min–max scaling method. All analyses were performed using MetaboAnalyst 5.0 (https://www.metaboanalyst.ca (accessed on 5 November 2024)).

## 3. Results and Discussion

### 3.1. Browning Index

**Figure 1:**
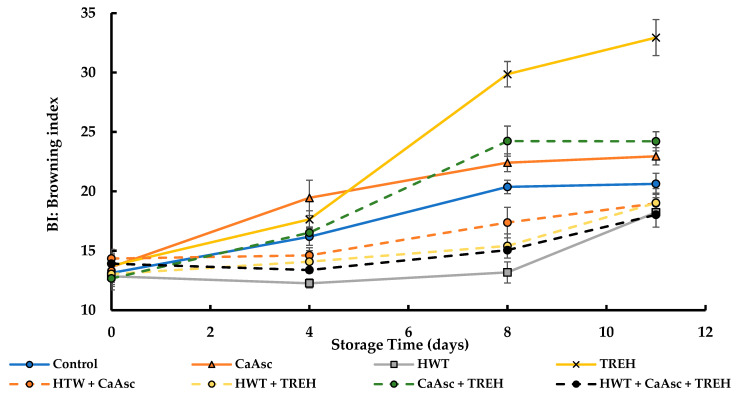
The browning index (BI) of the broccoli sticks during storage under different treatments. Data are presented as mean ± SEM (*n* = 10). ANOVA showed significant effects for main factors and their interaction. Fisher’s LSD test: LSD_Days x Treatment_ = 2.81, *p* < 0.05. CaAsc = 1% calcium ascorbate, TREH = 5% trehalose. HWT = hot water treatment at 55 °C for 1 min.

The evolution of BI in broccoli sticks across the different treatments during storage is shown in Figure 1. ANOVA revealed that both the storage time and the treatment had significant effects on the BI, with a strong interaction between these factors (*p* < 0.05). Notably, TREH, followed by CaAsc alone and the combination of both, exhibited the highest BI over time, even higher than the control. In contrast, HWT alone and its combinations led to only a slight increase in the BI, peaking at day 8, indicating that the treatment was effective in limiting browning compared to the control. The postharvest treatments proposed to mitigate this issue have distinct mechanisms and effects on enzymatic activity and subsequent browning. HWT at 55 °C for 1 min effectively reduced browning in the broccoli sticks, and this is likely due to the partial inactivation of PPO. Although PPO is considered to be thermostable in some plant species, moderate heat treatments can partially inactivate the enzyme, thereby slowing down enzymatic browning [26]. Similar results were observed in fresh-cut apples or melon peels, where HWT between 45 °C to 55 °C reduced browning and helped maintain the product [17,40]. Additionally, HWT is a non-chemical, easily applicable method for the fresh-cut industry that can effectively control enzymatic oxidation without negatively affecting sensory attributes.

CaAsc is a compound that combines calcium with ascorbic acid; the latter has well-known antioxidant properties. As mentioned in the introduction, ascorbic acid is widely used to inhibit enzymatic browning in fresh-cut vegetables due to its ability to reduce oxidised substrates back to their original phenolic forms, thereby preventing the formation of brown pigments. Ascorbic acid does not directly interact with the PPO enzyme but mitigates enzymatic browning by converting oxidised substrates back to their precursor diphenols [13]. Studies suggest that its anti-browning function is primarily attributed to its ability to reduce enzymatically formed o-quinones [41]. However, this effect may be less pronounced in certain fresh-cut fruits, such as pears [42], where ascorbic acid’s protective role diminishes once it is fully oxidised to dehydroascorbic acid. At this stage, o-quinones can no longer be reduced, allowing melanin formation to proceed and leading to continued browning. Similarly, in our study, no significant reduction in browning was observed in the broccoli sticks. This is likely due to the limited capacity of ascorbic acid at the studied concentration to sustain its reducing effect. This contrasts with what has been observed in fresh-cut pears, where browning intensifies once ascorbic acid is fully oxidised. In line with these findings, Xiong et al. [18] observed that the browning in fresh-cut potatoes treated with CaAsc (0.5% *w*/*v*) was quite similar to that in untreated samples. However, ascorbic acid applied at the same concentration proved highly effective in delaying both browning and wound healing, reducing reactive oxygen species (ROS) levels, and enhancing antioxidant capacity. This suggests that CaAsc presents a lower efficacy in decreasing browning in certain fresh-cut fruits, probably due to limited reductive activity. On the other hand, TREH has been shown to stabilise membranes and protect plant tissues from stress-induced damage [19]. However, in our experiment, its application led to an unexpected increase in browning. This phenomenon could be attributed to several factors. Firstly, TREH may have interacted with PPO and POD, stabilising these enzymes and prolonging their activity, thereby enhancing the conversion of phenolic compounds into o-quinones. Secondly, TREH may have interacted with the resulting quinones, intensifying browning, as sugars have been reported to promote quinone polymerisation and the formation of darker pigments [25]. Additionally, TREH may have influenced the tissue’s metabolic response, which may potentially have increased the respiration rate (as discussed in Section 3.3) and promoted the production of ROS, which could accelerate the oxidation of phenolic compounds. Unlike in litchi fruit (*Litchi chinensis*), where TREH (2.5 g/L) significantly reduced enzymatic browning by enhancing antioxidant enzyme activities and inhibiting PPO [24], in the broccoli sticks, it failed to prevent oxidative damage or delay browning, ultimately resulting in a more pronounced discolouration. These findings underscore the complexity of TREH’s impact on fresh-cut products and highlight the need for product-specific evaluations before being applied as a preservative.

### 3.2. Microbiological Analysis

**Figure 2:**
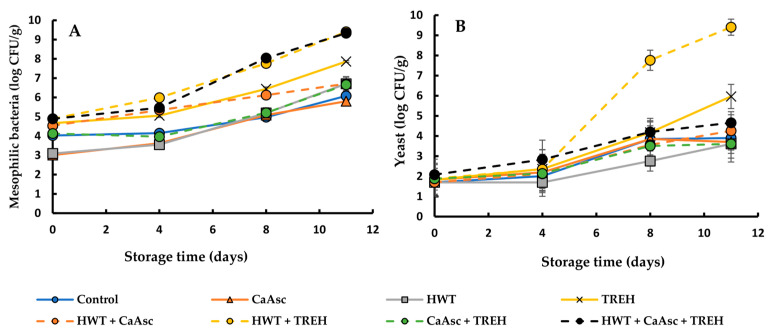
The growth of (**A**) mesophilic bacteria and (**B**) yeasts in the broccoli sticks during storage under different treatments. Data are presented as mean ± SEM (*n* = 3). ANOVA showed significant effects for main factors and their interaction. Fisher’s LSD test: LSD_Days x Treatment_ = 0.36 for bacteria and 0.34 for yeast, *p* < 0.05. CaAsc = 1% calcium ascorbate, TREH = 5% trehalose. HWT = hot water treatment at 55 °C for 1 min.

The evolution of total mesophilic aerobic bacteria and yeasts in the broccoli sticks under different treatments during storage is shown in Figure 2. ANOVA indicated that both the storage time and the treatment had significant effects on microbial growth (*p* < 0.05). From days 0 to 4, the mesophilic bacterial count remained below 4 log CFU/g in the CaAsc and HWT treatments. However, all the treatments, including the control, exhibited a progressive increase in bacterial populations, reaching 5.8 to 6.6 log CFU/g on day 11. In contrast, the TREH treatment, alone or in combination with HWT, showed the highest bacterial counts over time, reaching 7.7 to 9.4 log CFU/g on day 11, which may be related to enhanced microbial survival or proliferation; nevertheless, further studies are needed to confirm a direct effect.

Yeast counts remained stable during the initial 4 days but increased significantly with the storage time. From that day onwards, in particular, the HWT+TREH- and TREH-treated samples showed the highest yeast counts, reaching 9 log CFU/g and 6 log CFU/g, respectively, on day 11. Meanwhile, the rest of the treatments, the control included, showed lower yeast counts, ranging from 3.7 to 4.5 log CFU/g.

The combination of HWT + TREH proved to be the most favourable condition for microbial growth, leading to the highest counts of both mesophilic bacteria and yeasts. The enhanced microbial proliferation observed in the TREH-treated samples may be linked to the moisture-retaining capacity of TREH, which can modulate water activity in complex food matrices [25]. Additionally, certain bacteria and yeasts can metabolise TREH as an energy source, which could explain the increased microbial counts observed in those treatments. This effect was particularly pronounced when TREH was combined with HWT, as the heat may have enhanced nutrient dissolution and availability, further promoting microbial growth [43].

To explain the effect of CaAsc in the initial microbial control, it is important to consider the dual role of calcium and ascorbic acid in maintaining cell wall integrity and potentially influencing antimicrobial activity. Calcium reinforces cell walls, reducing pathogen penetration and enzymatic degradation, which can help limit microbial growth. Meanwhile, ascorbic acid may contribute to this effect by helping to maintain tissue integrity and the redox balance in the broccoli sticks, which could also play a role in microbial control [14,15,44]. On the other hand, HWT showed initial microbial control since heat may stimulate natural defence responses in plant tissues, enhancing resistance against microbial invasion [45] or it could delay enzymatic degradation by preserving cell wall integrity [28].

Pathogenic bacteria, including *Escherichia coli* and *Salmonella* spp., were analysed throughout the storage period, although no detectable counts were obtained (<2 log CFU/g). This underscores the effectiveness of the initial washing and disinfection steps in minimising microbial contamination and ensuring the microbiological safety of the broccoli sticks. Moreover, the absence of these pathogens meets the microbiological criteria established by Commission Regulation (EC) No 2073/2005 [46] for ready-to-eat foods, including minimally processed vegetables. Despite their absence, continued monitoring is crucial, as *E. coli* and *Salmonella* spp. remain key food safety indicators due to the potential risk they pose under favourable growth conditions. These findings are consistent with previous studies on fresh-cut produce, which highlight the role of rigorous hygiene practices and effective sanitation in controlling microbial proliferation [47].

### 3.3. Gas Composition Analysis

**Figure 3:**
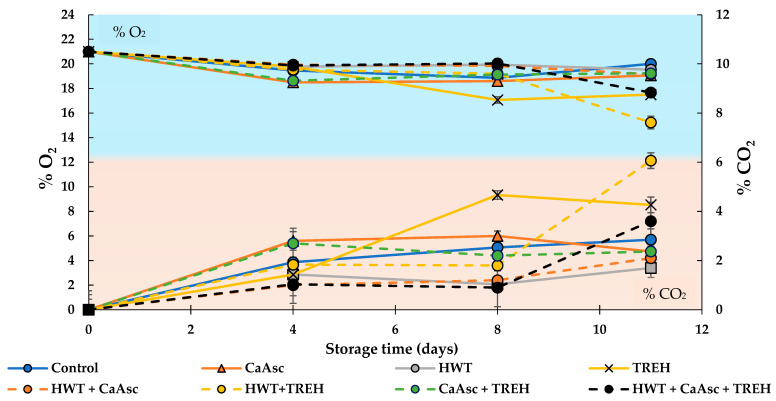
Changes in O_2_ and CO_2_ levels in the broccoli sticks during storage under different treatments. Data are presented as mean ± SEM (*n* = 3). ANOVA showed significant effects for the main factors and their interaction. Fisher’s LSD test: LSD_Days x Treatment_ = 0.55 for O_2_ and 0.66 for CO_2_, *p* < 0.05. CaAsc = 1% calcium ascorbate, TREH = 5% trehalose. HWT = hot water treatment at 55 °C for 1 min.

The treatments HWT + TREH, TREH, and HWT + CaAsc + TREH exhibited the highest CO_2_ concentrations inside the packaging by the end of storage, with values exceeding 3% and reaching up to 6%. Concurrently, the oxygen levels in these samples decreased to between 15% and 17%, indicative of elevated respiration activity (Figure 3). This slightly enhanced metabolic activity suggests a moderate increase in oxidative demand, which may be associated with accelerated senescence and microbial proliferation. In fact, the highest mesophilic bacterial counts were found in these treatments (Section 3.2). This factor could partially explain the higher CO_2_ levels, as microbial respiration contributes to CO_2_ production during growth [48]. However, the HWT treatment, without TREH, resulted in the lowest CO_2_ accumulation (<2%) and maintained higher O_2_ concentrations (around 19–20%), indicating reduced respiration rates and a slower metabolic activity. This behaviour is closely associated with delayed senescence and extended shelf-life in fresh-cut produce.

These findings reinforce the effectiveness of mild thermal treatments to modulate plant tissue respiration, as previously reported in citrus fruits, where HWT initially increased respiration but subsequently stabilised it, preserving postharvest quality with no adverse effects [49].

Moreover, the results underline the efficacy of HWT as a non-chemical strategy to maintain freshness and reduce spoilage during cold storage. Interestingly, the effects of TREH appear to be product-specific. In whole peppers and apples, TREH has been shown to suppress weight loss and reduce respiration rates, resulting in improved quality and extended shelf-life [50,51], which would suggest that the effectiveness of TREH treatment may depend on the type of product and its metabolic characteristics.

### 3.4. Sensory Analysis

**Figure 4:**
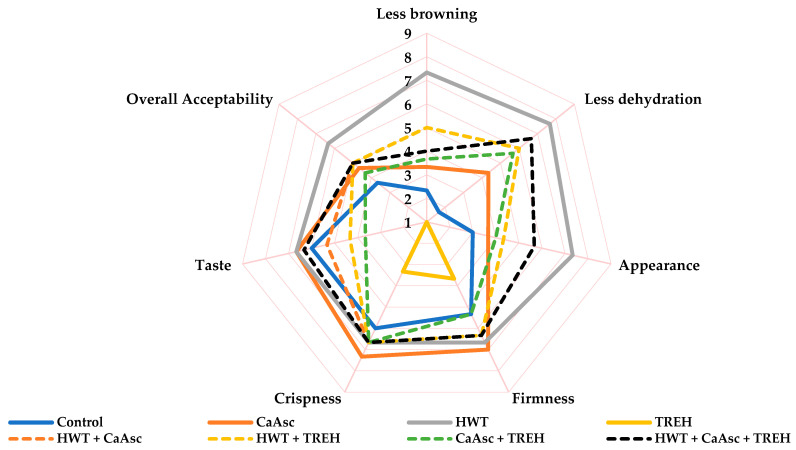
The sensory evaluation of the broccoli sticks on day 11 of storage under different treatments. Data are presented as mean values (*n* = 10). Data are based on a hedonic scale where 1 = unusable; 3 = poor; 5 = fair (limit of marketability); 7 = good; and 9 = excellent. ANOVA showed significant effects for the main factors and their interaction. Fisher’s LSD test: LSD_Days x Treatment_ = 0.55 for O_2_ and 0.66 for CO_2_, *p* < 0.05. CaAsc = 1% calcium ascorbate, TREH = 5% trehalose. HWT = hot water treatment at 55 °C for 1 min.

Figure 4 shows the sensory evaluation results of broccoli sticks on day 11 of storage under the different treatments. Appearance was strongly influenced by visual browning and dehydration. All the treatments, except for TREH, improved the visual appearance of broccoli sticks compared to the control. The most notable results were observed in the HWT treatment and its combinations, which consistently provided the lowest visual browning and dehydration. Regarding texture, crispness, and taste, the CaAsc and HWT treatments yielded the best results. In particular, the presence of calcium in the CaAsc treatment contributed to improved firmness and structural integrity, as previously demonstrated by other researchers [12,17]. The effect of HWT on maintaining firmness and improved appearance during storage has been reported for mandarin [49], fresh-cut apple, pear [52], and melon [16]. When considering overall sensory quality—integrating appearance, texture, and taste attributes—HWT emerged as the most effective treatment, extending the sensory shelf-life of the broccoli sticks to the 11-day limit.

**Figure 5:**
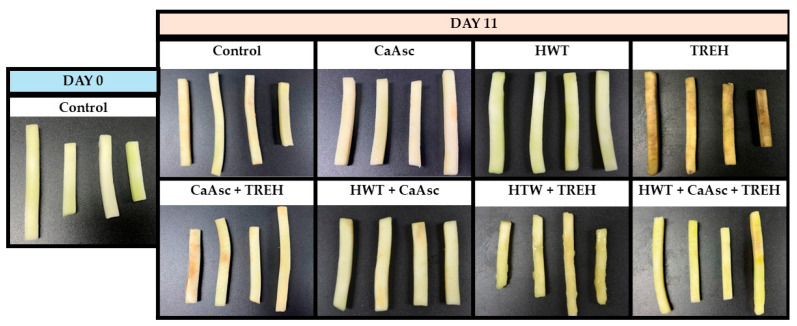
The visual appearance of the broccoli sticks: control sample on day 0 and samples under different treatments on day 11, included in the sensory analysis to assess visual changes over the storage period.

In contrast, the TREH treatment was the least effective, particularly in terms of browning and dehydration (Figure 5), where it received the lowest scores. TREH not only showed poor performance in these key attributes, which are crucial for consumer acceptance, but also in texture and appearance, highlighting its limitations as a standalone preservation method. These findings align with its inability to control microbial growth and prevent broccoli browning, as observed in this study. Interestingly, these results differ from previous research where TREH improved postharvest quality in other produce, such as peppers and guavas, by enhancing membrane stability, reducing water loss, and activating antioxidant enzymes such as peroxidase and catalase [21,51].

In line with these findings, TREH has been shown to improve firmness and water retention in cherry tomatoes [22] and kiwifruit [23]. The discrepancy in TREH’s effectiveness between broccoli and other produce comes from differences in cell structure, water content, and metabolic responses, which limit the capacity to maintain sensory quality in the broccoli sticks. Consequently, our findings emphasize that TREH alone is not suitable for preserving the sensory or microbial quality of broccoli sticks during storage. However, combination treatments with TREH showed an enhanced sensory quality compared to individual treatments. Notably, the HWT + CaAsc + TREH treatment achieved some of the best overall scores, particularly in taste, suggesting a positive combination that may enhance certain sensory attributes and increase the appeal of broccoli sticks to consumers after extended storage. Sensory parameters were monitored throughout storage (days 0, 4, 8, and 11) to evaluate the progression of quality over time. Detailed results can be found in the Supplementary Section (Figure S1).

### 3.5. Chemical Analysis

#### 3.5.1. Vitamin C Content

**Figure 6:**
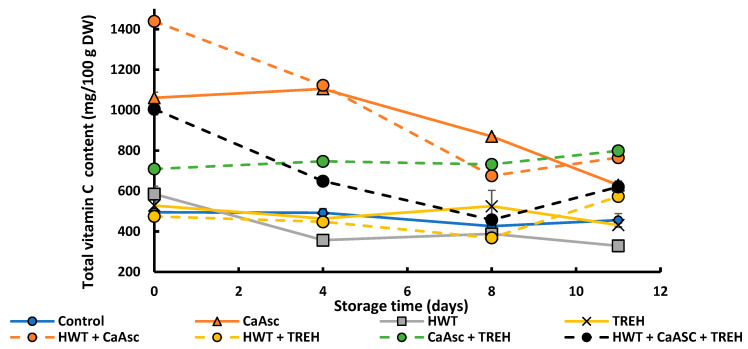
Total vitamin C content in the broccoli sticks during storage under different treatments. Data are presented as mean ± SEM (*n* = 3). ANOVA showed significant effects for main factors and their interaction. Fisher’s LSD test: LSD_Days x Treatment_ = 69.95, *p* < 0.05. CaAsc = 1% calcium ascorbate, TREH = 5% trehalose. HWT = hot water treatment at 55 °C for 1 min.

The changes in vitamin C content of the broccoli sticks are presented in Figure 6. At day 0, the HWT + CaAsc treatment exhibited the highest initial vitamin C content, reaching approximately 1500 mg/100 g DW, followed by the CaAsc treatment at around 1100 mg/100 g DW. As expected, immersion in a 1% CaAsc solution resulted in an increase in ascorbic acid content and, consequently, higher vitamin C. The enhanced response in HWT may be attributed to the thermal effect of HWT, which likely creates a gas expansion within the tissue that is followed by contraction during cooling, thereby promoting the absorption of the ascorbate solution (Figure S2). Furthermore, elevated temperatures may improve the diffusion rate of ascorbic acid and its solubility in both water and plant tissue. However, a gradual decline was observed with storage time in all the treatments that were combined with ascorbate. Interestingly, the CaAsc + TREH treatment showed a relatively stable vitamin C profile throughout storage, with minimal fluctuations. This suggests that the combination of CaAsc and TREH may contribute to ascorbic acid stabilisation, potentially due to TREH’s known ability to protect cellular structures and reduce oxidative degradation [23]. 

In this context, it would seem that TREH acts as a supportive agent, enhancing the protective effect of CaAsc rather than providing direct stabilisation on its own. This interpretation aligns with findings by Baek et al. [53], who reported the limited efficacy of TREH alone in preserving vitamin C in fresh-cut apples, thus emphasizing the dominant role of ascorbate. Therefore, the improved vitamin C retention observed in the CaAsc + TREH treatment can be attributed to a synergistic effect, where ascorbate serves as the primary antioxidant while TREH supports cellular integrity and reduces degradation pathways.

In contrast, the control, HWT, TREH, and HWT + TREH treatments exhibited a steady decline in vitamin C content over time. By day 11, CaAsc + TREH and HWT + CaAsc were among the treatments with the highest vitamin C levels, even surpassing the CaAsc only treatment. These findings indicate that CaAsc is the main driver of vitamin C enhancement and retention, and that its effectiveness may be maintained or even improved when combined with other treatments, depending on their interactions. The variability observed across treatments highlights the importance of optimising combined preservation strategies to balance compound protection with potential degradation mechanisms.

#### 3.5.2. Antioxidant Capacity and TPC

**Figure 7:**
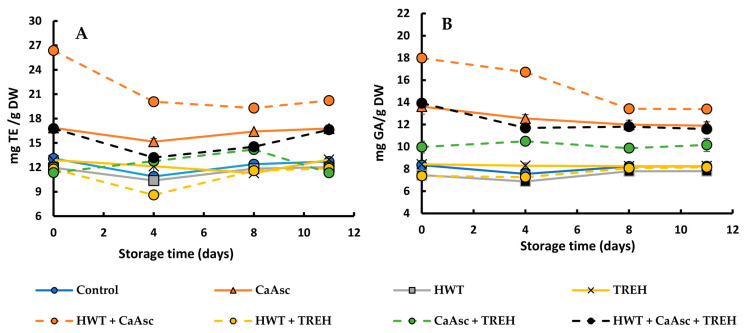
Changes in (**A**) antioxidant capacity and (**B**) TPC in the broccoli sticks during storage under different treatments. Data are presented as mean ± SEM (*n* = 3). ANOVA showed significant effects for the main factors and their interaction. Fisher’s LSD test: LSD_Days x Treatment_ = 0.76 for antioxidant capacity and 0.89 for total phenolic content, *p* < 0.05. CaAsc = 1% calcium ascorbate, TREH = 5% trehalose. HWT = hot water treatment at 55 °C for 1 min.

As shown in Figure 7, the treatments involving the application of CaAsc resulted in higher antioxidant capacity (A) and TPC (B) throughout storage, consistent with the trends observed for vitamin C levels (Figure 6).

The most pronounced effects were observed in the HWT + CaAsc treatment (27 mg TE/g and 17 mg GAE/g DW at day 0), followed by the CaAsc and HWT + CaAsc + TREH treatments, all of which maintained significantly higher antioxidant and phenolic values by the end of storage—even though HWT + CaAsc experienced a greater overall reduction of approximately 25%. The initial improvement in antioxidant preservation observed in treatments containing CaAsc is primarily attributed to the intrinsic activity of ascorbic acid and its protective role in stabilising phenolic compounds. Furthermore, when applied in combination with HWT, the uptake and efficacy of ascorbic acid appear to be enhanced, this is likely due to improved tissue permeability under mild thermal conditions, as previously discussed.

The CaAsc + TREH treatment resulted in the moderate preservation of both antioxidant capacity and TPC, with values falling to between those of the CaAsc-based treatments and the remaining ones. In contrast, the control, TREH, HWT + TREH, and HWT treatments exhibited consistently lower levels of antioxidant capacity and TPC, ranging from 11.3 to 13.0 mg TE/g and 7.4 to 8.4 mg GAE/g, respectively. These values remained relatively stable over time but were substantially lower throughout the storage period. The diminished antioxidant potential and TPC observed in these treatments is probably due to the absence of ascorbic acid from CaAsc, highlighting the central role of ascorbic acid in enhancing and preserving antioxidant compounds in the broccoli sticks during cold storage.

Previous studies have also highlighted the effectiveness of combining thermal strategies in fresh-cut vegetables to enhance the retention of bioactive compounds during storage [27]. In addition, Xiong et al. [18] reported that, although CaAsc was less effective than ascorbic acid and sodium isoascorbate in controlling browning and wound responses in fresh-cut potatoes, it did contribute to quality maintenance during storage. In particular, CaAsc treatment led to a greater accumulation of total phenolic and flavonoid contents, as well as enhanced antioxidant capacity compared to the control. These effects have been associated with reduced oxidative stress and better preservation of membrane integrity, which could contribute to the stabilisation of antioxidant systems during storage. Similarly, López-Hernández et al. [29] demonstrated that short HWT (e.g., 53 °C for 3 s) delayed oxidative damage in fresh-cut Chinese cabbage, improving the retention of antioxidant activity and TPC over time. In broccoli, Perini et al. [28] found that HWT applied to the basal part of the stems reduced the postharvest losses of polyphenols and helped maintain antioxidant capacity, emphasizing its role in extending shelf-life and preserving nutritional quality.

#### 3.5.3. Identification and Quantification of GSLs

**Figure 8:**
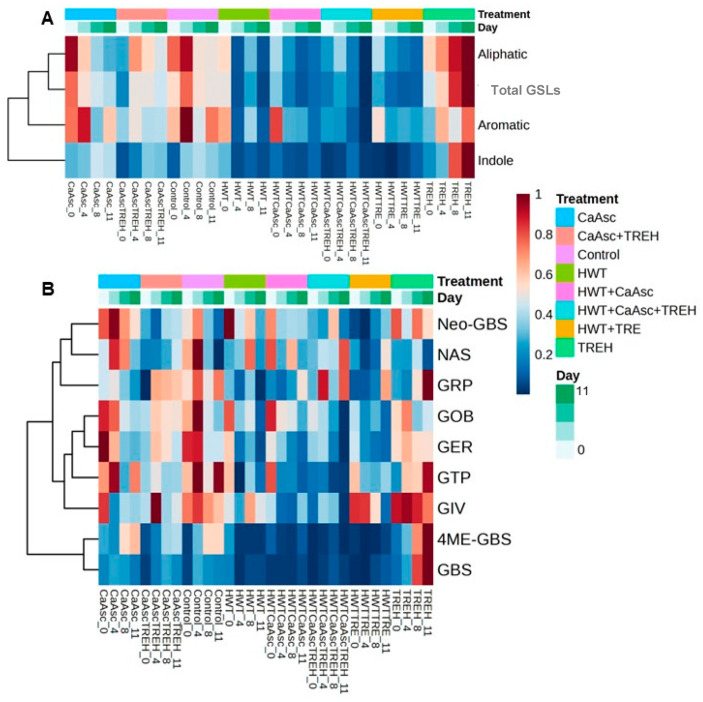
(**A**) Heatmap showing normalised levels of total, aliphatic, aromatic, and indole GSLs in the broccoli sticks stored at 5 ± 1 °C under different treatments (CaAsc = 1% calcium ascorbate, TREH = 5% trehalose. HWT = hot water treatment at 55 °C for 1 min). (**B**) Heatmap displaying normalised results of the quantification of individual GSLs: glucoraphanin (GRP), glucotropaeolin (GTP), glucoiberverin (GIV), glucoerucin (GER), glucobrassicin (GBS), neoglucobrassicin (NEO-GBS), glucoberteroin (GOB), 4-methoxyglucobrassicin (4ME-GBS), and gluconasturtiin (NAS) throughout storage. Values are shown on a colour scale from 0 to 1 after normalisation. Colour bars at the top indicate the treatments applied and the storage days (0, 4, 8, and 11). Data represent the mean of three independent replicates.

A total of nine GSLs from the *Brassicaceae* family were detected and comparatively analysed across all treatments and storage times. These included aliphatic GSLs, including glucoraphanin, glucoiberverin, glucoerucin, and glucoberteroin; aromatic GSLs, such as glucotropaeolin and gluconasturtiin; and indole GSLs, including glucobrassicin, neoglucobrassicin, and 4-methoxyglucobrassicin. Their relative abundance patterns after normalisation are given in Figure 8A,B; they reveal differences associated with treatment and storage time. The corresponding absolute quantification data are available in Supplementary Table S1.

The distribution of polyphenols and GSLs within broccoli stalk is uneven, with higher concentrations in the bark compared to the core, as reported by Costa-Pérez et al. [31]. These differences are related to the distinct physiological functions of each tissue and may also result from the disruption of tissue integrity caused by cutting, which can activate hydrolytic enzymes and modify the composition of secondary metabolites.

In our study, the absolute total GSL concentrations ranged from 79.05 ± 3.93 to 176.94 ± 7.37 mg/100 g DW (Supplementary Table S1), with aliphatic ones generally being the most abundant group, mainly glucoerucin as the main important, followed by indole and aromatic GSLs. Aliphatic GSLs, which dominated the overall GSL profile, showed high normalised values at day 0 in treatments such as TREH, CaAsc, and their combination, whilst the HWT treatments, particularly HWT + CaAsc and HWT + CaAsc + TREH, exhibited notably lower initial levels. Over time, most treatments showed a decrease in total GSL content; this was especially evident in HWT-based combinations.

Indole GSLs followed a similar trend, with higher values in early storage which progressively diminished, although treatments such as TREH and CaAsc + TREH showed relative preservation. Aromatic GSLs remained low across all treatments and storage times, with glucotropaeolin ranging from 2.51 ± 0.08 to 3.67 ± 0.07 mg/100 g DW, and gluconasturtiin from 0.98 ± 0.01 to 2.87 ± 0.40 mg/100 g DW.

Overall, these findings suggest that thermal treatments may reduce GSL content, while formulations containing TREH or CaAsc alone may better preserve GSLs during storage. Interestingly, the relative stabilisation or late increase in these compounds observed in the TREH-based treatments on day 11 could be linked to postharvest stress responses and tissue degradation. As reported by Paulsen et al. [54], mechanical damage from minimal processing may initially trigger GSL biosynthesis, while progressive senescence facilitates enzymatic hydrolysis due to the loss of cellular compartmentalisation, potentially altering GSL dynamics throughout storage.

Glucoerucin was initially detected at a higher concentration in the control and CaAsc-treated samples, ranging from 80.32 ± 1.06 mg/100 g to 87.16 ± 1.06 mg/100 g DW. When evaluating the interaction between storage time and treatment, significant differences were only observed in the TREH-treated samples, which exhibited an increase in the concentration of glucoerucin and glucoraphanin over time. In the case of glucoerucin, this upward trend was not observed when TREH was combined with HWT; however, the reduction was less pronounced than in the other treatments. In the case of glucoraphanin and glucotropaeolin, the TREH-treated samples showed a notable increase in their relative levels by day 11.

As no treatment-specific mechanism has been identified to directly explain this phenomenon, the observed increase may be attributable to microbial activity. Microorganisms are known to degrade cell membranes and, in some cases, produce myrosinase-like enzymes that facilitate the breakdown of GSLs into bioactive compounds. This hypothesis is supported by the significantly higher counts of mesophilic bacteria and yeasts observed in the TREH-treated samples at the end of storage (Figure 2), suggesting increased microbial metabolism. These observations are consistent with previous findings by Salas-Millán et al. [39] and Wu et al. [55], who demonstrated that lactic acid bacteria can enhance the release and transformation of GSLs in broccoli by-products, even in the absence of endogenous plant myrosinase.

The initial concentration of glucoiberverin was significantly lower in the HWT + CaAsc and HWT + CaAsc + TREH treatments, approximately half that found in the other treatments. This is consistent with the heatmap results, where those two treatments exhibited the lowest normalised values. In contrast, the remaining treatments, particularly TREH, HWT + TREH, and the control, displayed higher initial concentrations, reflected by higher normalised values. No significant interaction effects were observed, as all treatments showed a decreasing trend in glucoiberverin concentration over time.

The most notable differences in gluconasturtiin concentration were observed in the treatments involving CaAsc and HWT, either alone or in combination. While the overall detected levels of gluconasturtiin were low, a decreasing trend was observed in the control throughout storage. In contrast, most treated samples maintained stable levels over time, and an increase was observed at the end of the storage period in samples treated with the HWT + CaAsc + TREH combination. This mirrors the trend observed for glucoraphanin, suggesting a possible synergistic effect.

Glucoberteroin was detected at the lowest concentration among all quantified GSLs, representing less than 1% of the total GSL content (Table S1). Interestingly, its initial levels were slightly higher in samples subjected to HWT, CaAsc, and their combination (HWT + CaAsc), although a consistent decrease was observed across all treatments over time. These findings align with observations by Mbudu et al. [56], who reported tissue-specific variability of glucosinolates in kohlrabi tissues.

For indole GSLs such as glucobrassicin, 4-methoxyglucobrassicin, and neoglucobrassicin, the initial concentrations were not markedly higher in any particular treatment, but a gradual decrease over time was observed across most treatments. Notably, treatments containing TREH—especially alone—showed a relative increase in glucobrassicin by day 11, which may be associated with microbial activity, as signs of spoilage (e.g., increased CFU/g counts) were evident from day 8 onwards. A similar association was reported by Salas-Millán et al. [32], where the spontaneous fermentation of broccoli stalks led to a notable rise in indole GSLs, including glucobrassicin and 4-methoxyglucobrassicin, in parallel with the growth of lactic acid bacteria and yeasts. Additionally, as highlighted by Li et al. [3], microbial metabolism can modulate the profile of bioactive compounds in broccoli, particularly under stress or degradation conditions. These findings suggest that, beyond the direct effect of the treatments applied, microbial interactions during storage may play a role in the late-stage accumulation or transformation of specific GSLs. These findings are consistent with the results reported by Núñez-Gómez et al. [5], who found that indole GSLs in broccoli stalks—such as glucobrassicin, 4-methoxyglucobrassicin, and neoglucobrassicin—were less stable during extraction, and were only detected in the extractable fraction, unlike more stable aliphatic GSLs such as glucoraphanin.

## 4. Conclusions

This study demonstrates the potential of up-cycling broccoli stalks, a commonly discarded by-product, into a high-value fresh-cut product through the application of targeted postharvest strategies. The main conclusions are as follows:

(a) Among the treatments evaluated, HWT alone was highly effective in reducing browning, a key factor in achieving an 11-day shelf-life, whilst also controlling microbial growth and respiration and yielding the highest scores in sensory quality. However, its ability to preserve bioactive compounds, particularly GSLs, was limited.

(b) To retain and enhance these health-promoting compounds, the HWT + CaAsc treatment was the most effective, primarily due to the antioxidant action of ascorbic acid present in CaAsc. This treatment significantly increased vitamin C retention (by 78%), antioxidant capacity (by 68%), and TPC (by 65%) compared to the control on day 11.

(c) Although the control treatment, based solely on peracetic acid disinfection, provided microbial reduction, the inclusion of antioxidant support in other combined treatments helped to maintain sensory quality and played a key role in stabilising vitamin C and reducing the degradation of both phenolic compounds and GSLs.

(d) Conversely, TREH applied alone did not contribute to extending the shelf-life. It was associated with enhanced tissue browning, elevated respiration rates, and increased microbial proliferation, factors potentially associated with membrane destabilisation and, consequently, the enhancement of GSL bioavailability. These results underscore the crop-specific nature of postharvest responses, as TREH, despite its reported benefits, did not perform consistently in this matrix. Future studies should explore the effect of different concentrations in cruciferous tissues.

(e) The absence of foodborne pathogens such as *Salmonella* spp. and *E. coli* confirms the efficacy of the initial sanitation and preservation procedures in ensuring microbiological safety throughout the storage time.

Overall, these findings highlight the value of integrated postharvest strategies, particularly those combining mild heat and ascorbate-based antioxidant dips, for preserving the quality and bioactive compounds in fresh-cut broccoli. The HWT + CaAsc treatment proved to be the most balanced option: it maintained sensory attributes and enhanced the retention of health-promoting compounds and achieved a shelf-life of 11 days at 5 °C under MAP. Given that both components are safe, commonly used, and cost-effective, this approach offers a scalable and sustainable solution for broccoli stalk valorisation in the fresh-cut industry. This approach not only supports food loss reduction and sustainable processing but also represents a viable up-cycling strategy aligned with circular economy principles. Future research should investigate the physiological mechanisms underlying these effects and evaluate their applicability to other vegetable by-products, contributing to loss and waste valorisation and the development of functional fresh-cut products.

## Data Availability

The original contributions presented in this study are included in the article. Further inquiries can be directed to the corresponding author.

## Supplementary Materials

The following supporting information can be downloaded at: https://www.mdpi.com/article/10.3390/foods14142476/s1. Figure S1: Sensory evaluation of the broccoli sticks subjected to different treatments: (A) Control, (B) CaAsc, (C) HWT, (D) TREH, (E) CaAsc+TREH, (F) HWT+CaAsc, (G) HWT+TREH, and (H) HWT+CaAsc+TREH. Each radar chart shows the evolution of sensory attributes during storage (days 0, 4, 8, and 11). The evaluated parameters included overall acceptability, taste, crispness, firmness, appearance, less dehydration, and less browning. Data are expressed as mean values (n = 10), using a 9-point hedonic scale where 1 = unusable, 3 = poor, 5 = fair (limit of marketability), 7 = good, and 9 = excellent. CaAsc = 1% calcium ascorbate, TREH = 5% trehalose, HWT = hot water treatment at 55 °C for 1 min. Figure S2: Schematic representation of the proposed synergistic mechanisms of the combined hot water treatment (HWT, 55 °C for 1 min) and calcium ascorbate dip (CaAsc, 1%, 5 °C for 1 min) applied to fresh-cut broccoli stalks. HWT induces a thermal effect that creates gas expansion within the tissue; subsequent cooling in a chilled CaAsc solution causes tissue contraction; this sequence promotes the absorption of the CaAsc solution, enhancing antioxidant protection. Table S1:Glucosinolate contents (mg/100 g dry weight) in broccoli sticks during storage under different treatments.
