# Supplementary material for: Up-Cycling Broccoli Stalks into Fresh-Cut Sticks: Postharvest Strategies for Quality and Shelf-Life Enhancement
Source: Foods. 2025 Jul 15;14(14):2476. doi: 10.3390/foods14142476 (PMC12294733; doi:10.3390/foods14142476)
Supplement: Supplementary file 1 [file foods-14-02476-s001.zip › foods-3713454-supplementary.pdf]

foods

MDPI

Supplementary Materials

Up-Cycling Broccoli Stalks into Fresh-Cut Sticks: Postharvest Strategies for Quality and Shelf-Life Enhancement

Nieves García-Lorca [1,2], José Ángel Salas-Millán [2] and Encarna Aguayo [1,2,*]

[1] Postharvest and Refrigeration Group, Universidad Politécnica de Cartagena (UPCT), Paseo Alfonso XIII, 48, 30203 Cartagena, Spain; nieves.garcial@upct.es
[2] Food Quality and Health Group, Institute of Plant Biotechnology (IBV-UPCT), Campus Muralla Del Mar, R&D Building, 30202 Cartagena, Spain; joseangel.salas@upct.es
* Correspondence: encarna.aguayo@upct.es

https://doi.org/10.3390/foods14142476

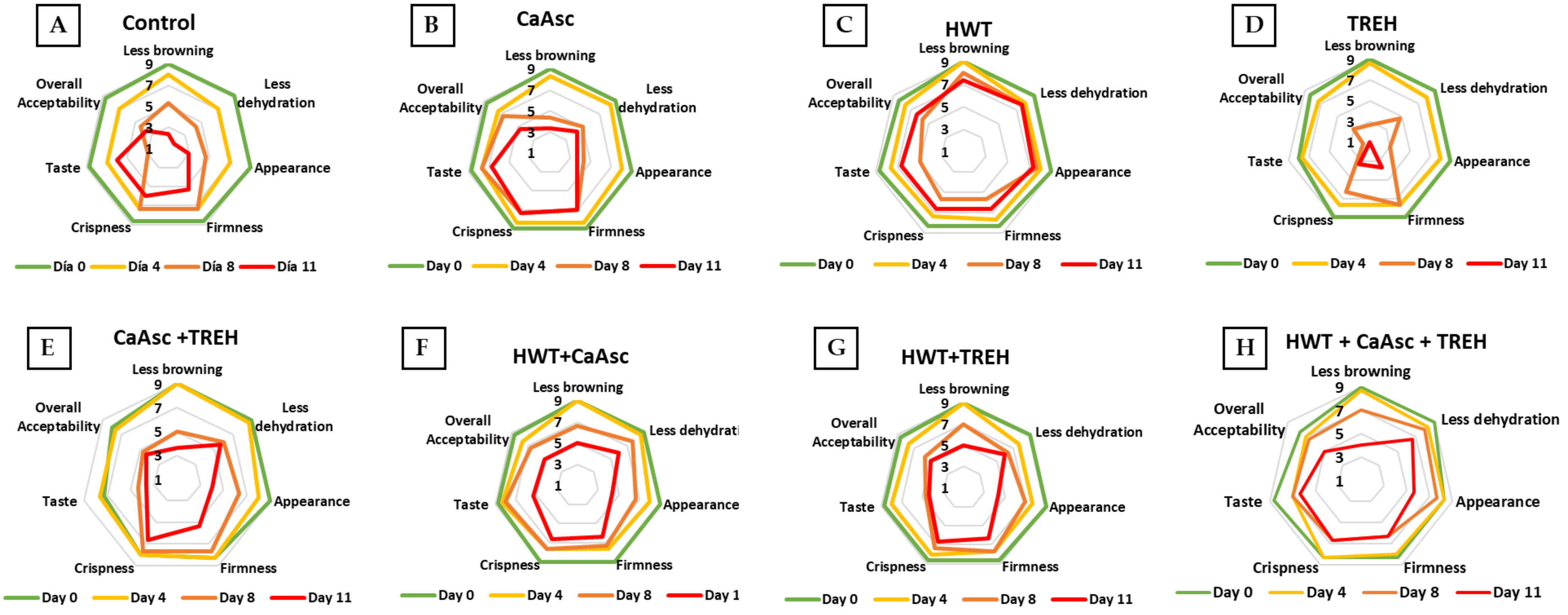

Figure S1. Sensory evaluation of the broccoli sticks subjected to different treatments: (A) Control, (B) CaAsc, (C) HWT, (D) TREH, (E) CaAsc+TREH, (F) HWT+CaAsc, (G) HWT+TREH, and (H) HWT+CaAsc+TREH. Each radar chart shows the evolution of sensory attributes during storage (days 0, 4, 8, and 11). The evaluated parameters included overall acceptability, taste, crispness, firmness, appearance, less dehydration, and less browning. Data are expressed as mean values (n = 10), using a 9-point hedonic scale where 1 = unusable, 3 = poor, 5 = fair (limit of marketability), 7 = good, and 9 = excellent. CaAsc = 1% calcium ascorbate, TREH = 5% trehalose, HWT = hot water treatment at 55 °C for 1 min.

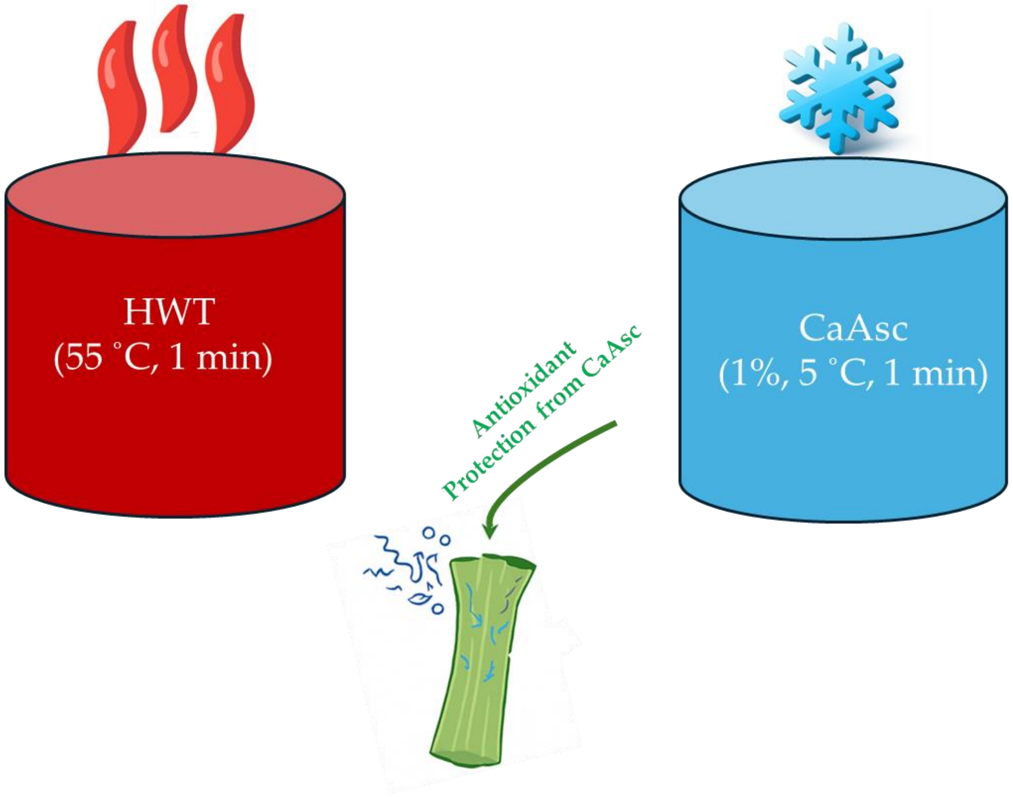

Figure S2. Schematic representation of the proposed synergistic mechanisms of the combined hot water treatment (HWT, 55 °C for 1 min) and calcium ascorbate dip (CaAsc, 1%, 5 °C for 1 min) applied to fresh-cut broccoli stalks. HWT induces a thermal effect that creates gas expansion within the tissue; subsequent cooling in a chilled CaAsc solution causes tissue contraction; this sequence promotes the absorption of the CaAsc solution, enhancing antioxidant protection.

Table S1. Glucosinolate contents (mg/100 g dry weight) in broccoli sticks during storage under different treatments.

Treatment	Storage time	Aliphatic				Aromatic		Indole			Σ GSLs
		GRP	GIV	GER	GOB	GTP	NAS	GBS	4ME-GBS	NEO-GBS	
Control	0	6.85[z] ± 0.11	9.43 ± 0.71	80.32 ± 1.55	1.11 ± 0.11	3.23 ± 0.39	2.21 ± 0.20	6.54 ± 0.05	11.99 ± 0.42	11.21 ± 0.71	132.90 ± 8.29
	4	6.58 ± 0.41	9.66 ± 0.61	81.20 ± 4.44	1.34 ± 0.01	3.67 ± 0.07	2.87 ± 0.40	13.29 ± 0.39	20.14 ± 0.17	12.79 ± 0.20	154.32 ± 8.25
	8	8.38 ± 0.09	8.87 ± 0.15	60.11 ± 1.74	0.99 ± 0.03	3.09 ± 0.28	1.58 ± 0.20	11.62 ± 0.35	27.90 ± 1.05	9.88 ± 0.50	130.62 ± 6.31
	11	8.11 ± 0.62	7.87 ± 1.14	60.03 ± 0.47	0.91 ± 0.12	3.65 ± 0.14	0.98 ± 0.01	10.46 ± 0.25	27.75 ± 3.14	8.42 ± 0.22	128.17 ± 6.31
CaAsc	0	6.33 ± 0.31	10.73 ± 0.05	87.16 ± 1.06	1.25 ± 0.12	3.31 ± 0.33	1.82 ± 0.17	10.46 ± 0.52	18.93 ± 0.67	13.30 ± 0.85	153.29 ± 8.98
	4	6.60 ± 0.45	5.95 ± 0.16	69.52 ± 1.83	1.20 ± 0.04	3.61 ± 0.12	1.60 ± 0.39	12.56 ± 0.12	17.10 ± 0.16	14.64 ± 0.35	130.77 ± 7.14
	8	5.68 ± 0.07	5.32 ± 0.20	54.73 ± 3.44	0.99 ± 0.07	2.83 ± 0.15	1.34 ± 0.71	12.07 ± 0.28	27.64 ± 0.80	12.54 ± 1.44	123.14 ± 5.80
	11	5.04 ± 0.16	4.85 ± 0.34	53.03 ± 2.38	0.94 ± 0.06	3.36 ± 0.21	1.58 ± 0.13	9.58 ± 0.36	29.62 ± 0.58	11.34 ± 0.40	119.35 ± 5.76
HWT	0	5.57 ± 0.38	6.25 ± 0.48	67.10 ± 3.55	1.20 ± 0.08	3.21 ± 0.07	1.50 ± 0.16	10.41 ± 0.10	17.93 ± 0.13	14.81 ± 0.07	127.98 ± 6.89
	4	4.31 ± 0.06	3.52 ± 0.15	46.01 ± 0.84	0.92 ± 0.04	2.51 ± 0.08	1.62 ± 0.05	5.41 ± 0.05	11.75 ± 0.61	10.78 ± 0.03	86.82 ± 4.72
	8	5.77 ± 0.21	8.67 ± 0.32	52.98 ± 1.96	1.00 ± 0.03	2.93 ± 0.14	1.42 ± 0.19	6.61 ± 0.45	11.81 ± 0.24	11.87 ± 0.69	104.07 ± 5.34
	11	3.79 ± 0.38	6.22 ± 0.52	41.88 ± 2.51	0.76 ± 0.04	2.67 ± 0.08	1.41 ± 0.09	6.61 ± 0.70	11.56 ± 0.83	9.57 ± 0.72	84.46 ± 4.24
TREH	0	5.47 ± 0.05	10.92 ± 0.06	56.30 ± 2.16	1.00 ± 0.07	2.68 ± 0.11	1.56 ± 0.15	5.14 ± 0.26	11.13 ± 0.56	9.60 ± 0.41	94.81 ± 5.84
	4	4.77 ± 0.41	12.08 ± 0.90	69.51 ± 1.41	1.16 ± 0.03	3.21 ± 0.05	1.43 ± 0.20	12.67 ± 0.54	20.04 ± 0.25	10.70 ± 0.66	135.56 ± 7.12
	8	6.81 ± 0.25	11.14 ± 0.68	64.74 ± 2.18	0.91 ± 0.14	3.18 ± 0.11	1.79 ± 0.17	29.55 ± 1.25	32.26 ± 1.14	12.95 ± 0.38	163.33 ± 6.97
	11	9.66 ± 0.11	9.53 ± 1.00	64.40 ± 2.08	0.98 ± 0.09	3.60 ± 0.18	1.14 ± 0.03	34.87 ± 1.76	41.20 ± 1.76	11.56 ± 0.89	176.94 ± 7.37
HWT + CaASC	0	5.22 ± 0.37	11.50 ± 0.71	64.88 ± 4.42	1.05 ± 0.09	2.76 ± 0.27	1.44 ± 0.35	8.69 ± 0.21	15.31 ± 0.33	13.29 ± 0.42	124.13 ± 6.62
	4	4.35 ± 0.24	10.96 ± 0.25	56.13 ± 0.87	1.02 ± 0.08	2.73 ± 0.07	1.51 ± 0.31	5.83 ± 0.19	11.95 ± 0.59	10.27 ± 0.57	104.74 ± 5.83
	8	5.07 ± 0.48	8.75 ± 0.07	47.57 ± 1.25	1.00 ± 0.09	2.73 ± 0.10	1.20 ± 0.10	5.65 ± 0.10	11.73 ± 0.14	10.44 ± 0.45	94.11 ± 4.89
	11	6.77 ± 0.96	4.63 ± 0.78	44.58 ± 0.82	0.87 ± 0.08	2.62 ± 0.06	1.49 ± 0.05	6.74 ± 0.05	13.53 ± 0.89	10.32 ± 0.95	91.56 ± 4.52
HWT + TREH	0	4.85 ± 0.29	5.57 ± 0.10	62.99 ± 2.60	1.25 ± 0.04	3.43 ± 0.08	1.49 ± 0.18	5.83 ± 0.18	13.84 ± 0.15	12.67 ± 0.60	112.93 ± 6.47
	4	4.18 ± 0.21	1.72 ± 0.48	48.68 ± 3.37	0.83 ± 0.08	2.87 ± 0.31	1.08 ± 0.24	4.90 ± 0.03	10.74 ± 0.67	7.51 ±0.37	91.51 ± 4.97
	8	4.68 ± 0.12	1.98 ± 0.24	45.64 ± 0.54	0.88 ± 0.12	2.84 ± 0.20	1.21 ± 0.17	6.67 ± 0.31	10.93 ± 0.74	8.92 ± 0.12	88.77 ± 4.61
	11	7.70 ± 0.14	1.97 ± 0.32	44.19 ± 2.81	0.77 ± 0.04	2.94 ± 0.20	1.96 ± 0.05	7.74 ±0.07	12.27 ± 0.41	10.02 ± 0.38	89.55 ± 4.49
CaASC + TREH	0	4.17 ± 0.18	10.81 ± 0.57	60.37 ± 4.74	0.96 ± 0.06	3.21 ±0.06	1.45 ± 0.29	5.21 ± 0.21	11.61 ± 0.87	7.97 ± 0.30	105.76 ± 6.21
	4	7.63 ± 0.74	12.69 ± 0.49	67.99 ± 1.55	1.07 ± 0.05	3.07 ± 0.12	1.31 ± 0.30	9.82 ± 0.35	15.82 ± 0.27	8.35 ± 0.12	127.76 ± 6.93
	8	7.42 ± 0.43	5.04 ± 0.38	64.50 ± 2.13	1.04 ± 0.08	2.93 ± 0.04	1.49 ± 0.29	12.84 ± 0.49	22.48 ± 0.65	10.40 ± 0.28	128.14 ± 6.67
	11	7.26 ± 0.19	6.18 ± 0.45	59.68 ± 1.54	1.03 ± 0.04	2.95 ± 0.17	1.82 ± 0.09	10.91 ± 0.50	23.16 ± 0.73	10.55 ± 0.06	123.55 ± 6.16
HWT+ CaAsc + TREH	0	3.65 ± 0.16	5.44 ± 0.50	55.55 ± 1.01	0.95 ± 0.02	2.93 ± 0.02	1.29 ± 0.20	6.02 ± 0.12	12.90 ± 0.34	9.55 ± 0.32	98.28 ± 5.73
	4	8.99 ± 0.16	5.00 ± 0.26	57.76 ± 3.45	0.95 ± 0.06	2.70 ± 0.05	1.79 ± 0.33	6.18 ± 0.92	11.79 ± 0.63	9.26 ± 0.34	104.43 ± 5.90
	8	5.73 ± 0.06	4.38 ± 0.23	46.72 ± 0.50	0.85 ± 0.01	2.56 ± 0.10	1.75 ± 0.21	7.33 ± 0.29	12.93 ± 0.32	11.96 ± 0.16	94.23 ± 4.75
	11	8.40 ± 0.39	0.34 ± 0.01	38.55 ± 0.88	0.69 ± 0.04	2.55 ± 0.09	2.49 ± 0.08	6.24 ± 0.45	11.06 ± 0.25	8.73 ± 0.43	79.05 ± 3.93
LSD-Treatments (T)		0.50	0.71	3.36	0.10	0.23	0.35	0.10	0.52	0.76	4.77
LSD-Days of storage (D)		0.35	0.50	2.38	0.07	NS	NS	0.07	0.73	0.54	3.37
LSD interaction: T X D		1.00	1.42	6.72	0.20	0.47	0.70	0.20	1.46	1.53	9.53

[z]Mean values ± SE (n = 3). The individual glucosinolates quantified include glucoraphanin (GRP), glucotropaeolin (GTP), glucoiberverin (GIV), glucoerucin (GER), glucobrassicin (GBS), neoglucobrassicin (NEO-GBS), glucoberteroin (GOB), 4-methoxyglucobrassicin (4ME-GBS), and gluconasturtiin (NAS). Total glucosinolate content (Σ GSLs) represents the

sum of all quantified glucosinolates. ANOVA showed significant effects for the main factors and their interaction. Fisher's LSD test was used for multiple comparisons: treatments (T), storage time (D), and their interaction (T × D), $p < 0.05$. ASC = 1% calcium ascorbate, TREH = 5% trehalose. HWT = hot water treatment at 55 °C for 1 min.
